# Kidney function is associated with plasma ATN biomarkers among Hispanics/Latinos: SOL-INCA and HCHS/SOL results

**DOI:** 10.1186/s13195-025-01786-8

**Published:** 2025-06-19

**Authors:** Natasha Z. Anita, Wassim Tarraf, Sayaka Kuwayama, Freddie Márquez, Charles DeCarli, Bharat Thyagarajan, Nora Franceschini, James P. Lash, Tanya Johns, Kevin A. González, Martha Daviglus, Haibo Zhou, Ariana M. Stickel, Frank J. Penedo, Tatjana Rundek, Doug Galasko, Hector M. González

**Affiliations:** 1 Department of Neurosciences, University of California, San Diego, 9500 Gilman Dr, La Jolla, San Diego, CA USA; 2 Institute of Gerontology & Department of Healthcare Sciences, Wayne State University, Detroit, MI USA; 3 Department of Neurology, University of California, Davis, Sacramento, CA USA; 4 Department of Laboratory Medicine and Pathology, University of Minnesota, Minneapolis, MN USA; 5 Department of Epidemiology, Gillings School of Public Health, University of North Carolina at Chapel Hill, Chapel Hill, NC USA; 6 Department of Medicine, University of Illinois Chicago, Chicago, IL USA; 7 Department of Medicine, Division of Nephrology, Albert Einstein College of Medicine/Montefiore Medical Center, Bronx, NY USA; 8 Institute for Minority Health Research, College of Medicine, University of Illinois Chicago, Chicago, IL USA; 9 Department of Biostatistics, University of North Carolina at Chapel Hill, Chapel Hill, NC USA; 10 Department of Psychology, San Diego State University, San Diego, CA USA; 11 Departments of Psychology and Medicine, University of Miami, Coral Gables, USA; 12 Department of Neurology and Evelyn F. McKnight Brain Institute, University of Miami, Miami, FL USA

**Keywords:** Kidney, Renal, Alzheimer's disease, Dementia, Biomarkers

## Abstract

**Background:**

Plasma amyloid-tau-neurodegeneration (ATN) biomarker levels may be influenced by non-brain systems, such as kidney function, which could impact the interpretation of ATN biomarker results, particularly in groups like Hispanic/Latino individuals with higher rates of cardiometabolic health issues. Here, we examine the association between kidney function and plasma ATN markers among a diverse sample of Hispanic/Latino individuals living in the U.S.

**Methods:**

Data was collected from the Hispanic Community Health Study/Study of Latinos (HCHS/SOL, Visit 1, 2008–2011), the largest prospective cohort study of noninstitutionalized Hispanic/Latino adults in the U.S., and its ancillary study, the Study of Latinos-Investigation of Neurocognitive Aging (SOL-INCA) which was conducted during the second visit of the parent HCHS/SOL study (Visit 2, 2015–2018). SOL-INCA aimed to examine the neurocognitive decline of middle-aged and older Hispanic/Latino adults, and the inclusion criteria were the age of 50-years and older by Visit 2 and completion of battery of neurocognitive tests at Visit 1. Survey linear regression models were used to examine associations between CKD status (estimated glomerular filtration rate [eGFR] < 60 ml/min/1.73m^2^ or urine albumin-creatinine ratio [uACR]) > = 30 mg/g) and the plasma ATN biomarkers (β-amyloid 42/40 ratio [Aβ42/40 ratio], phosphorylated-tau181 [p-Tau181], neurofilament light [NfL], and glial fibrillary associated protein [GFAP]), independently. All models adjusted for sociodemographic and cardiometabolic factors (BMI, diabetes, and hypertension).

**Results:**

5,968 participants were included in the study (mean age 63.4 ± 8.1, 54% women). CKD was associated with higher p-Tau181 (b = 0.82), NfL (b = 11.60) and GFAP levels (b = 31.41), and lower Aβ42/Aβ40 ratio (b=-0.004). Lower eGFR (i.e., reduced kidney function) was associated with higher p-Tau181, NfL, and GFAP levels (b ranges [-0.87 - -0.03]), and lower Aβ42/Aβ40 ratio (b = 0.000). Higher (natural log) uACR was associated with lower Aβ42/Aβ40 ratio and higher levels of all other biomarkers (b ranges [0.24–5.49]). Additionally, CKD, eGFR, and uACR were associated with ATN biomarkers in models adjusted for cardiometabolic risk factors, diabetes and hypertension.

**Conclusions:**

CKD status, kidney function and urinary markers of kidney damage are significant confounders in the interpretation of plasma ATN biomarker levels.

**Supplementary Information:**

The online version contains supplementary material available at 10.1186/s13195-025-01786-8.

## Background

Biomarkers of Alzheimer’s Disease (AD) – β-amyloid, tau and neurodegeneration (ATN) as biological evidence for diagnosing AD etiology in vivo – have garnered wide interest in the field [1–5]. Historically, AD diagnosis was based primarily on clinical symptoms, particularly progressive memory impairment and functional decline. However, advances in biomarker research have led to a paradigm shift toward biological definitions of the disease. In 2018, the National Institute on Aging and Alzheimer’s Association (NIA-AA) proposed the ATN research framework, shifting the definition of AD from a syndromic classification based on clinical symptoms to one rooted in biological markers [2]. This framework categorizes biomarkers into three groups: amyloid-β deposition (A), tau pathology (T), and neurodegeneration (N), and has since provided a foundation for studies exploring biomarker-driven diagnosis and staging of AD. The recent 2024 Alzheimer’s Association Working Group revised criteria build upon this model by incorporating both biological and clinical dimensions, underscoring the importance of aligning plasma biomarker research with these updated diagnostic standards [3]. The revised criteria also included inflammatory (I) markers (e.g., glial fibrillary acidic protein [GFAP]) to reflect the broader pathophysiological heterogeneity of AD. This evolving conceptualization supports a potential shift toward an AT(N)I framework, which may enhance disease characterization and risk stratification, particularly in high-risk groups such as individuals with kidney dysfunction.

Given the growing body of research and the field’s transition from cerebrospinal fluid biomarkers to less invasive blood-based biomarkers for clinical and research use, it is essential to identify bodily systems that may impact the accurate interpretation of biomarker levels. The kidney system, specifically chronic kidney disease (CKD), has been identified as one system failure that is related to ATN blood-based biomarkers levels [6–8]. These findings also suggest that kidney function and other unexplored cardiometabolic functions may influence ATN blood-based biomarkers levels thereby affecting the interpretation of results.

Interpreting blood-based biomarkers levels for groups with excess cardiometabolic health problems, including Hispanics/Latinos, is critically important to avoid potential misinterpretation of biomarker results and diagnostic errors. Notably, Hispanic/Latino individuals experience excess diabetes and hypertension, which frequently go undetected and untreated with consequent kidney dysfunction and disease [9–11]. Accordingly, we postulate that kidney dysfunction will be linked to plasma ATN biomarker changes.

Here, we examine kidney function in relation to plasma-based ATN biomarkers β-amyloid (Aβ), phosphorylated tau 181 (p-Tau-181), neurofilament light chain (NfL) – indicative of amyloidosis, tauopathy, and non-specific neurodegeneration, respectively – in a large and representative sample of Hispanic/Latino individuals, the *Study of Latinos-Investigation of Neurocognitive Aging* (SOL-INCA). GFAP, a marker of astrocytic activation and neuroinflammation, was also evaluated due to its growing recognition as a potential biomarker in dementia research [3, 12].

This study addresses critical gaps in the literature by focusing on underrepresented groups, particularly Hispanic/Latino individuals, who are disproportionately impacted by both AD [13] and chronic kidney disease (CKD) [9, 14, 15]. To date, several studies have demonstrated associations between kidney dysfunction and ATN blood-based biomarkers [6–[8, 16], but they have been limited to predominantly non-Hispanic White populations, leaving significant questions about the generalizability of these findings to non-White populations. Our study also includes a comprehensive panel of blood-based biomarkers, including those for neurodegeneration and astrocytic activation, and has the potential to inform more inclusive diagnostic and monitoring strategies for AD and CKD.

We hypothesize that primary risk factors for kidney dysfunction (diabetes and hypertension) will interact with kidney function measures in relation to ATN biomarkers. The study findings will provide evidence that cardiometabolic risk factors and kidney dysfunction play essential roles in the expression of ATN blood-based biomarkers among Hispanic/Latino individuals.

## Methods

### Data

We used data from the Hispanic Community Health Study/ Study of Latinos (HCHS/SOL) and its ancillary study, the Study of Latinos-Investigation of Neurocognitive Aging (SOL-INCA; 2015–2018) [17]. HCHS/SOL is the largest prospective cohort study of noninstitutionalized Hispanic/Latino adults in the U.S., designed to examine health risks and disease burden in this population. At the baseline visit of HCHS/SOL (Visit 1; 2008–2011), *N* = 16,415 self-identified Hispanic/Latino adults ages 18–74 were probability sampled using complex survey design (clustering, stratification) from four U.S. metropolitan cities densely populated with individuals with diverse Hispanic/Latino backgrounds (Bronx, NY; Chicago IL, Miami, FL; San Diego, CA). Detailed description of the study objectives, design and sampling methods are published elsewhere [18, 19]. SOL-INCA is an ancillary study conducted during the second visit of the parent HCHS/SOL study (Visit 2; 2014–2017). Inclusion criteria for SOL-INCA were participants aged 50 years or older at the time of HCHS/SOL Visit 2 and had completed the neurocognitive testing battery during HCHS/SOL Visit 1; however, neurocognitive test results were not used in the present analysis. *N* = 6,377 participants were enrolled, consented, and completed the SOL-INCA visit (i.e., second HCHS/SOL visit). The HCHS/SOL Coordinating Center generated probability weights specific for each study visit to allow inference to the target population, and these weights were incorporated in all analyses. Biomarker data and all covariates, except for sex and background, were measured at HCHS/SOL Visit 2 (see Covariates section below for more information). The Institutional Review Board approved the study protocol at all sites, and the participants provided informed written consent.

### Plasma biomarker outcomes

At each HCHS/SOL field centers, fasting blood samples were drawn through a venipuncture method (55–70 mL) by trained phlebotomists. Blood tubes were mixed with the anticoagulant (EDTA) to prevent clotting within 15 min of collection and were centrifuged at 3,000 x g for 30 min at 15 °C. Subsequently, plasma was withdrawn, aliquoted, and placed in the − 70 °C freezer. Frozen plasma samples were packaged in freezer storage bags and shipped to the Central Laboratory at the University of Minnesota for future analysis [20].

Using these samples, all plasma biomarkers were measured (pg/mL) using the highly sensitive Simoa (single molecule array) HD-X analyzer (Quanterix; Billerica, MA). A specific assay kit to assay plasma Aβ40, Aβ42, NfL, and GFAP was the Simoa Neurology 4-Plex E Advantage (multiplex; product #103670). A subset of plasma NfL samples were assessed using the Simoa NF-light Advantage (singleplex; product #103186), and the plasma p-Tau181 was assayed with the Simoa p-Tau181 Advantage v2 (product #103714) [21]. The Aβ42/40 ratio was also calculated. Extreme and censored values of the plasma ATN biomarkers were excluded from the analyses to minimize the impact of outliers influencing estimates. In sensitivity analyses, biomarkers values below or above 3 standard deviations from the weighted, target population, mean were also excluded from analyses.

### Exposures

At HCHS/SOL Visit 2, serum creatinine (mg/dL) and cystatin C (mg/L) were both measured on a Roche Modular P Chemistry Analyzer (Roche Diagnostics Corporation, Indianapolis, IN), using a creatinine enzymatic method and a turbidimetric method, respectively. Additionally at Visit 2, a spot urine sample was collected, and urine microalbumin (mg/dL) and urine creatinine (mg/dL) were assayed respectively using an immunoturbidometric method on the ProSpec nephelometric analyzer (Dade Behring GMBH. Marburg, Germany) and using a creatinase enzymatic method on a Roche Modular P Chemistry Analyzer (Roche Diagnostics Corporation, Indianapolis, IN) [22].

Based on these data, we measured (1) the estimated glomerular filtration rate (eGFR; Chronic Kidney Disease Epidemiology Collaboration creatinine-cystatin C age, sex 2021 Eq. (23)), and (2) the urine albumin-creatinine ratio (uACR: urine microalbumin/urine creatinine).

Our primary exposure is a binary indicator of chronic kidney disease (CKD [23]; no, yes) at Visit 2, defined as having either (1) a low eGFR (< 60 ml/min per 1.73 m2) or (2) albuminuria (uACR > = 30 mg/g). In our secondary analysis, we operationalize eGFR and uACR (natural log-transformed due to a skewed distribution) continuously as indicators of kidney functions.

### Covariates

We included age (years), sex (female, male), Hispanic/Latino background (Dominican, Central American, Cuban, Mexican, Puerto Rican, South American, other), apolipoprotein E genotypes (*APOE*; ε2/ε2, ε2/ε3, ε2/ε4, ε3/ε3, ε3/ε4, ε4/ε4), body mass index (BMI; underweight < 18.5 kg/m^2^, normal 18.5–24.9 kg/m^2^, overweight 25.0–29.9 kg/m^2^, obese > = 30 kg/m^2^; WHO criteria [24]), diabetes (normal glucose regulation, impaired glucose tolerance, diabetes; ADA criteria [25]), and hypertension (no, yes; NHANES criteria [26]).

The indicators of diabetes and hypertension were first operationalized continuously (glycosylated hemoglobin percentage [HbA1c], and systolic and diastolic blood pressure) and in subsequent models, categorically (using ADA and NHANES criteria, respectively). All covariates, except for sex and background, were measured at Visit 2.

### Statistical analyses

First, we report the descriptive characteristics of the SOL-INCA target population, overall and by the CKD status. The mean differences for the continuous measures by the CKD status are tested using F-tests, and the distributional differences of the categorical indicators by the CKD status are tested using chi-square tests.

Second, in primary analyses, to investigate the associations of CKD with plasma ATN biomarkers (Aβ42/40, p-Tau181, NfL, and GFAP) independently, we fit survey linear regression models with two sets of covariates adjustments. The first model (M1) included age, sex, background, *APOE*, BMI, HbA1c, systolic and diastolic blood pressure. The second model (M2) adjusted the same set of covariates as M1, except for the diabetes and hypertension indicators in place for HbA1c, systolic and diastolic blood pressure.

Third, we investigated whether the associations of CKD with the plasma ATN biomarkers are modified by diabetes and hypertension. To do so, we used the covariate adjusted model (M2) described above (diabetes and hypertension categorically operationalized) and included interaction terms of diabetes and CKD, and hypertension and CKD.

In secondary analyses, we used the survey linear regressions models to examine the relationship between our secondary exposures measuring kidney function (continuous measures of eGFR and natural log uACR modeled independently) and the plasma ATN biomarkers. We used the same covariate adjustments as the main analysis reported above (M1, M2).

Lastly, to assess robustness of the results from the primary analyses, we conduct the following sensitivity analyses: (1) examine the associations of low eGFR (eGFR < 60 ml/min per 1.73 m2; prevalence = 6.7%) and albuminuria (uACR > = 30 mg/g; prevalence = 13.8%) with plasma ATN biomarkers independently; (2) examine the associations of CKD and NfL using multiplex assays only (*n* = 5,791); (3) examine the associations of CKD with plasma ATN biomarkers while excluding values of the plasma ATN biomarkers at 3 standard deviations below and above the mean. In all sensitivity analyses, we used the same set of covariates included in the original analyses (M1, M2).

In post-hoc analysis, we estimated and plotted the average marginal means from models above, by the level of CKD (no, yes) and the continuum of the continuous exposures (eGFR and uACR) to help facilitate interpretation of the results.

## Results

### Target population characteristics

The characteristics are reported in Table 1. Out of the *N* = 6,377 SOL-INCA participants, *N* = 6,226 participated in plasma specimen collection. We excluded *N* = 258 who had missing data for the covariates for an analytic sample of *N* = 5,968. However, the available sample size for the ATN outcomes after exclusion varied between markers (unweighted *n* = 5,478, *n* = 5,686, *n* = 5,880 and *n* = 5,709 for Aβ42/40, p-Tau181, NfL, and GFAP, respectively). Average age was 63.4 years, and 54% were female. Roughly 80% met criteria for overweight (40%) and obese (42%), approximately 58% were hypertensive, 36% met ADA diabetes criteria. The average values for eGFR and uACR were 92.7 ml/min/1.73m^2^ and 63.4 mg/g, respectively. The prevalence of low eGFR (eGFR < 60 ml/min per 1.73 m^2^) and albuminuria (uACR > = 30 mg/g) were 6.7% and 13.8%, respectively. The distribution of these variables differed by the CKD status.

**Table 1:** Descriptive statistics of the overall *Study of Latinos – Investigation of neurocognitive aging* (SOL-INCA) target population and by chronic kidney disease (CKD) status

	No CKD (Unweighted *N* = 5041, 82.8%)	CKD (Unweighted *N* = 927, 17.2%)	Total (Unweighted *N* = 5968, 100%)	*P*-value
	% (SE)	% (SE)	% (SE)	
**Sex**				
Female	55.3 (1.0)	48.2 (2.4)	54.1 (0.9)	0.009
Male	44.7 (1.0)	51.8 (2.4)	45.9 (0.9)	
**Hispanic Background**				
Dominican	9.8 (0.8)	7.2 (1.1)	9.4 (0.8)	< 0.001
Central American	7.5 (0.6)	6.0 (1.0)	7.3 (0.6)	
Cuban	25.7 (2.0)	29.5 (3.3)	26.4 (1.9)	
Mexican	33.9 (1.8)	27.2 (2.5)	32.8 (1.7)	
Puerto Rican	13.4 (0.8)	24.2 (2.1)	15.2 (0.8)	
South American	5.7 (0.4)	3.5 (0.7)	5.3 (0.4)	
Other	4.0 (0.5)	2.4 (0.9)	3.7 (0.5)	
**Body Mass Index**				
Underweight	0.5 (0.1)	0.5 (0.3)	0.5 (0.1)	< 0.001
Normal	17.6 (0.8)	12.7 (1.5)	16.8 (0.7)	
Overweight	41.4 (1.1)	36.0 (2.4)	40.4 (1.0)	
Obese	40.5 (1.0)	50.8 (2.5)	42.3 (1.0)	
**Hypertension**				
No	46.8 (1.0)	18.0 (1.6)	41.8 (0.9)	< 0.001
Yes	53.2 (1.0)	82.0 (1.6)	58.2 (0.9)	
**Diabetes**				
Normal glucose regulation	18.1 (0.8)	7.6 (1.0)	16.3 (0.7)	< 0.001
Impaired glucose tolerance	51.4 (1.0)	32.1 (2.3)	48.1 (1.0)	
Diabetes	30.5 (1.0)	60.3 (2.3)	35.6 (1.0)	
**APOE Alleles**				
2/2	0.3 (0.1)	0.0 (0.0)	0.3 (0.1)	0.704
2/3	7.2 (0.5)	7.2 (1.1)	7.2 (0.4)	
2/4	1.1 (0.2)	1.5 (0.6)	1.2 (0.2)	
3/3	70.4 (0.9)	70.5 (1.9)	70.4 (0.8)	
3/4	19.4 (0.8)	19.6 (1.8)	19.4 (0.7)	
4/4	1.6 (0.2)	1.1 (0.5)	1.5 (0.2)	
	**Mean (SD)**	**Mean (SD)**	**Mean (SD)**	**P-value**
**Age**	62.6 (8.0)	67.1 (8.0)	63.4 (8.1)	< 0.001
**Body Mass Index**	29.5 (5.2)	30.8 (6.0)	29.7 (5.4)	< 0.001
**Glycosylated hemoglobin %**	6.1 (1.2)	7.1 (1.9)	6.3 (1.4)	< 0.001
**Systolic blood pressure**	128.3 (17.8)	140.0 (20.0)	130.3 (18.8)	< 0.001
**Diastolic Blood pressure**	72.9 (10.4)	73.7 (11.3)	73.0 (10.6)	0.259
**uACR**	5.8 (5.5)	340.4 (1135.8)	63.4 (511.8)	< 0.001
**ln(uACR)**	1.4 (0.8)	4.2 (1.6)	1.9 (1.5)	< 0.001
**eGFR**	96.7 (15.8)	73.6 (26.8)	92.7 (20.4)	< 0.001

Notes: Unweighted sample sizes (N) are reported. All percentages and other estimates are weighted to account for the complex survey design of the SOL-INCA study. The distributional differences by the CKD status were tested using survey adjusted chi-squared tests and F-tests for categorical and continuous measures respectively. All covariates were measured at Visit 2, except for sex and Hispanic/Latino background assessed at Visit 1. Exposures were measured at Visit 2

*Abbreviations*: CKD = chronic kidney disease, M = Mean, SD = Standard deviation, SE = Standard error, eGFR = estimated glomerular, filtration rate, uACR = urine albumin-creatinine ratio

### Primary analysis – associations between CKD and plasma ATN biomarkers

**Fig. 1:**
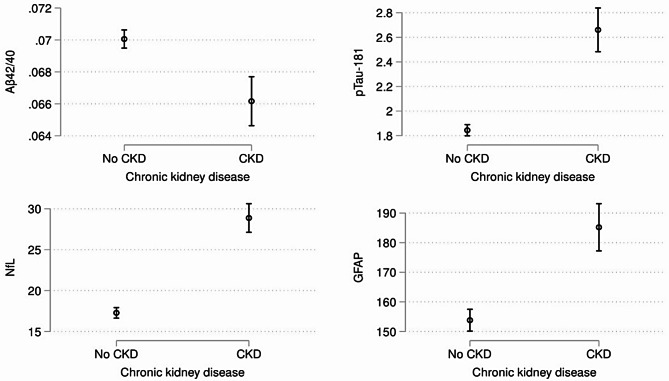
Associations of chronic kidney disease (CKD) with plasma biomarkers in *Study of Latino–Investigation of Neurocognitive Aging*. *Note*. Aβ = beta-amyloid, pTau = phosphorylated tau, NfL = neurofilament light, GFAP = glial fibrillary acidic protein. The model adjusted for age, sex, Hispanic/Latino background, apolipoprotein E (APOE) genotype, body mass index, diabetes, and hypertension

The estimates are reported in Table 2. In the model adjusted for age, sex, background, *APOE*, BMI, HbA1c, systolic and diastolic blood pressure (M1), having CKD was associated with lower Aβ42/40, and elevated p-Tau181, NfL, and GFAP (b[CI 95%]_Aβ42/40_ =-0.003[-0.005; -0.002]; b[CI 95%]_p−Tau181_ =0.774[0.587; 0.960], b[CI 95%]_NfL_ = 10.307[8.475; 12.139], b[CI 95%]_GFAP_ = 29.202[20.512; 37.892]; ps < 0.001). These associations were consistent when using the alternative covariates adjustment including diabetes and hypertension indicators (M2). The estimated average marginal means for each of the plasma ATN biomarkers by the CKD groups (no, yes) are presented in Fig. 1.

**Table 2:** Associations of chronic kidney disease with plasma ATN biomarkers in the *Study of Latinos – Investigation of neurocognitive Aging*, estimated using survey-weighted linear regression models

	CKD	
	M1	M2
	b [CI 95%]	b [CI 95%]
Aβ42/40	-0.003*** [-0.005;-0.002]	-0.004*** [-0.006;-0.002]
pTau-181	0.774*** [0.587;0.960]	0.816*** [0.631;1.002]
NfL	10.307*** [8.475;12.139]	11.601*** [9.662;13.539]
GFAP	29.202*** [20.512;37.892]	31.405*** [22.748;40.061]

*Notes*: All reported values account for the complex survey designs of the SOL-INCA. Each of the plasma biomarker outcomes was independently modeled as a function of chronic kidney disease (CKD). M1 adjusted for age, sex, Hispanic/Latino background, apolipoprotein E genotype (APOE), body mass index, glycosylated hemoglobin, systolic blood pressure, and diastolic blood pressure. M2 adjusted for age, sex, Hispanic/Latino background, APOE, body mass index, diabetes, and hypertension

*Abbreviations*: Aβ = beta-amyloid, pTau = phosphorylated tau, NfL = neurofilament light, GFAP = glial fibrillary acidic protein, b = beta, CI = confidence intervals

^*^p < 0.05, ^**^p < 0.01, ^***^p < 0.001

### Interaction analysis – interactions between diabetes/hypertension and CKD on plasma ATN biomarkers

**Fig. 2:**
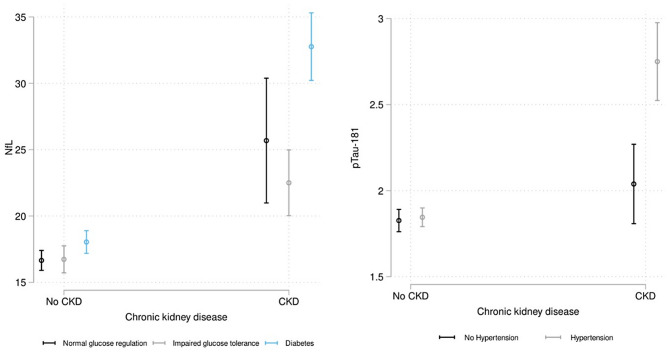
Associations of chronic kidney disease (CKD) with plasma biomarkers in the Study of Latinos – Investigation of Neurocognitive Aging; interaction by A) diabetes (NfL) and B) hypertension (pTau-181). Note. NfL=neurofilament light, pTau=phosphorylated tau. The model adjusted for age, sex, Hispanic/Latino background, apolipoprotein E (APOE) genotype, body mass index, diabetes, and hypertension, and included the interaction terms of CKD and diabetes as well as CKD and hypertension

The test statistics for the interaction terms are presented in Table 3. We found evidence to support modifications by diabetes status in the associations of CKD with NfL(F[2,611] = 8.86; *p* < 0.001), whereby the presence of CKD amplified the effect of diabetes on NfL levels. In other words, individuals with CKD and diabetes exhibited higher NfL levels compared to individuals with diabetes alone. The estimated marginal means by diabetes groups are presented in Fig. 2 to illustrate the differential associations of CKD and NfL by the diabetes groups (normal glucose regulation, impaired glucose tolerance, diabetes).

We also found evidence that hypertension modifies the associations of CKD with p-Tau181(F[1,612] = 17.2; p = < 0.001); namely having CKD had more pronounced effects in people with hypertension. Specifically, individuals with CKD and hypertension reported higher pTau-181 levels compared to individuals with hypertension alone. The estimated marginal means by the hypertension groups (no, yes) were also estimated and presented in Fig. 2.

**Table 3:** Associations of chronic kidney disease with plasma biomarkers in the *Study of Latinos – Investigation of neurocognitive Aging*; interaction by diabetes and hypertension

Diabetes				
Outcome	F-test	*P*-value	df1	df2
Aβ42/40	0.034	0.967	2	607
pTau-181	1.586	0.205	2	611
NfL	8.855	**0.000**	2	611
GFAP	1.601	0.203	2	609
Hypertension				
Aβ42/40	1.894	0.169	1	608
pTau-181	17.170	**0.000**	1	612
NfL	3.328	0.069	1	612
GFAP	1.401	0.237	1	610

Note. All reported values account for the complex survey designs of the SOL-INCA. Each of the plasma biomarker outcomes was independently modeled as a function of chronic kidney disease (CKD). The model adjusted for age, sex, Hispanic/Latino background, apolipoprotein E genotype (APOE), body mass index, diabetes, and hypertension, and included the interaction terms of CKD and diabetes as well as CKD and hypertension

*Abbreviations*: Aβ = beta-amyloid, pTau = phosphorylated tau, NfL = neurofilament light, GFAP = glial fibrillary acidic protein, df = degree of freedom

### Secondary analysis – associations between continuous kidney measures and plasma ATN biomarkers

The results from the secondary analysis examining the associations between kidney functions measured continuously (eGFR and uACR) and the plasma ATN biomarkers are reported in Additional File 1. Higher eGFR (denoting better kidney function) was positively associated with Aβ42/40 (b[95% CI] = 0.000[0.000;0.000]), and negatively with other outcomes (b[CI 95%]_p−Tau181_ =-0.026[-0.031; -0.022], b[CI 95%]_NfL_ = -0.335[-0.383; -0.287], b[CI 95%]_GFAP_ = -0.840[-0.988; -0.692]; ps < 0.001) in M1. These results are consistent when using the model adjustment M2.

Higher (natural log) uACR (indicating kidney damage) was negatively associated with Aβ42/40 (b[95% CI] = -0.001[-0.001;-0.000], *p* < 0.01), and positively with other outcomes (b[CI 95%]_p−Tau181_ =0.235[0.173; 0.297], b[CI 95%]_NfL_ = 3.038 [2.402; 3.673], b[CI 95%]_GFAP_ = 4.736 [2.248; 7.224]; ps < 0.01) in M1 as well as M2. The estimated marginal means across the continuum of the eGFR and uACR are plotted along with their 95% confidence intervales in Additional File 1.

### Sensitivity analysis

Results from all three sensitivity analyses showed no substantive alterations of the findings reported through the primary analyses above. All corresponding results from these sensitivity models are reported in Additional File 1.

## Discussion

In a large representative community-based cohort of middle-aged and older Hispanic/Latino individuals, we found that CKD was associated with higher levels of p-Tau181, NfL, and GFAP, and lower Aβ42/Aβ40 ratio. Higher levels of kidney dysfunction (i.e., lower plasma eGFR and higher uACR) were associated with higher levels of p-Tau181, NfL, and GFAP, and lower Aβ42/Aβ40 ratio. Lastly, the associations between kidney function measures and plasma Aβ42/Aβ40 ratio, NfL, p-Tau181, and GFAP were independent of primary cardiometabolic risk factors for kidney dysfunction (diabetes and hypertension, data not shown). Together, these results suggest that decreased kidney clearance are associated with increased levels of these plasma biomarkers (i.e., p-Tau181, NfL, and GFAP; and impaired clearance of Aβ42 compared to Aβ40). Since eGFR remains the best marker for renal clearance, it is important to establish ATN biomarker reference levels specifically for individuals with low eGFR. This distinction ensures that elevated biomarker levels are interpreted correctly, avoiding misattribution to neuropathology.

The kidneys contribute to biomarker elimination through glomerular filtration and tubular secretion, processes influenced by characteristics such as molecule size, polarity, and protein binding. Biomarkers such as Aβ40 and Aβ42 fall near the upper threshold of what can be filtered, making them susceptible to accumulation when kidney function declines [27]. Notably, the Aβ42/40 ratio appears more resistant to these renal effects in the current study, potentially offering a more reliable marker in populations with reduced eGFR. Additionally, CKD-associated interstitial injury, tubular dysfunction, and systemic inflammation may further reduce clearance of blood-based biomarkers, contributing to peripheral accumulation [28]. Although these elevations may not reflect increased brain biomarker burden, they could confound the interpretation of these biomarkers. Moreover, CKD introduces other confounding factors, such as oxidative stress, vascular calcification, and hyperhomocysteinemia, that are independently associated with cognitive impairment [29–31]. Together, these mechanisms highlight the complexity of interpreting ATN biomarker levels in individuals with impaired kidney function and underscore the need for caution when using plasma biomarkers for AD diagnosis in CKD populations.

The association between CKD and higher p-Tau181 levels is consistent with the Mayo Clinic Study on Aging (MCSA) [7], although that study did not examine the other biomarkers included in our work. The associations between eGFR and both p-Tau181 and NfL are in line with a previous study conducted in older adults [6]. The cutoff values of amyloid-β positron emission tomography (Aβ-PET) positivity have been shown to differ for both p-Tau181 and NfL in individuals with mild to moderate eGFR decline compared to those with normal eGFR [32], suggesting that adjustments may be required for the use of diagnostic biomarkers even in individuals with mild kidney decline. Similarly, Lehmann and colleagues found that while p-Tau181 predicted conversion to dementia and detected Aβ positivity in older adults with mild cognitive impairment, the diagnostic cut-offs were affected by kidney status as defined by creatinine and/or eGFR [33]. In another study, excluding participants with CKD affected the plasma p-Tau181 and p-Tau217 cut-offs for detecting Aβ-PET [7], suggesting that kidney function may affect p-Tau levels regardless of isoform. The differences in p-Tau181 and p-Tau 217 associated with CKD were more pronounced and exceeded those observed between Aβ-PET-positive and Aβ-PET-negative groups [7], underscoring the importance of considering kidney function.

Stocker and colleagues reported sex-specific associations between eGFR and GFAP, finding these associations only in males [6]. Previous research has shown that GFAP levels tend to be higher in females [34–37], however we did not perform sex-stratified analyses in the current study as no differences were found between males and females. Sex differences have also been reported for other blood ATN biomarkers, where women had higher p-Tau-181 levels and lower Aβ42/Aβ40 ratios compared to men [38]. Future studies in the current population should conduct stratified analyses to determine potential sex-specific associations between kidney measures and ATN biomarkers. Although biomarker levels did not differ by sex in the current study, this does not preclude the possibility of sex-related differences in their associations with kidney measures. We employed linear regressions controlling for several covariables a priori, including sex, to determine associations between kidney function and plasma ATN biomarkers; other studies did not control for these same covariables [6, 36, 38]. Other important distinctions between our study and previous studies of blood ATN biomarkers are the population and study design. Stocker et al. conducted a nested case-control study with participants diagnosed with AD between baseline and a 17-year follow-up, including those with incident vascular dementia and mixed dementia diagnoses, and randomly selected controls. The population was also primarily White [6]. Rosano et al. examined a Caribbean population of African ancestry without information on cognitive status [38]. Conversely, our study included a broader Hispanic/Latino population with varying cognitive statuses, including those with mild to moderate cognitive impairment reported at the follow-up visit (average follow-up time of 7 years). Several studies have reported significant associations between kidney markers and cognitive function [39–45], and between blood ATN biomarkers and cognitive function [46–49]. Given the strong association between CKD severity and cognitive impairment, adjusting for cognitive function in all analyses may provide a more comprehensive understanding of the relationship between CKD and blood ATN biomarkers in future studies. Further research is needed to determine whether kidney function affects levels of blood-based ATN biomarkers in a way that contributes to accelerated cognitive decline and increased risk of dementia.

Our findings between higher eGFR and lower uACR with higher plasma Aβ42/Aβ40 ratios contrast with a previous study in the Alzheimer’s Disease Neuroimaging Initiative (ADNI) population, which reported no associations between eGFR and plasma Aβ42/Aβ40 [32]. In the MCSA population, individuals with CKD had higher plasma Aβ42/Aβ40 ratios compared to those without CKD [50]; however, in our study CKD was associated with lower ratios. These discrepancies may be due to differences in study populations, as both ADNI and MCSA populations were predominantly White and on average 10-years older than our SOL-INCA cohort. Further work is needed to elucidate whether age and ethnicity may be potential modifiers on the relationship between kidney function and plasma Aβ42/Aβ40 ratios.

Diabetes and hypertension, primary risk factors for kidney dysfunction, showed significant interactions with CKD in relation to NfL and p-Tau-181 in the current study. Specifically, the presence of CKD intensified the association between diabetes and elevated NfL levels, and between hypertension and higher p-Tau-181 levels. Diabetes status has previously been associated with higher plasma NfL levels in non-Hispanic Whites [51]. No associations were found between diabetes status and total tau or Aβ42/Aβ40 in that study, which is consistent with our findings. With regards to hypertension, VandeBunte et al. [52] previously reported a significant association between higher systolic blood pressure and elevated plasma p-Tau-181 levels, while no associations were found for Aβ42/Aβ40, NfL, or GFAP, consistent with our findings. Interestingly, that study reported significant associations between higher pulse pressure and higher levels of pTau181, NfL, and GFAP. Our current study utilized NHANES criteria to define hypertension, and did not account for pulse pressure, which represents a potential direction for future research. No significant associations were found between primary risk factors for kidney dysfunction and GFAP or Aβ42/Aβ40 in our study, indicating that these ATN biomarkers may not be as affected by diabetes or hypertension status. These results suggest that reference ranges for NfL and p-Tau-181 may need to be adjusted for diabetes and hypertension, respectively, in addition to CKD.

This study has several strengths. To the best of our knowledge, this is the largest biomarker study utilizing a probability-based (i.e., representative) sample. A key strength of our study is its focus on the preclinical stage of AD, as the cohort primarily consists of individuals under the age of 65. This characteristic enhances our contribution to the ongoing efforts in preclinical AD biomarker research by providing valuable insights into early disease markers within a representative population. We have included the largest, well-characterized community-based and representative sample of diverse Hispanic/Latino individuals living in four U.S. metropolitan areas (Bronx, NY; Chicago IL, Miami, FL; San Diego, CA) [17]. Identifying influencing factors for plasma ATN biomarkers is crucial as blood-based markers gain traction in the dementia field. Conducting these studies in Hispanic/Latino individuals is particularly important, as this group is significantly underrepresented in neurocognitive aging and biomarker research despite being the largest minority population in the U.S [17]. eGFR was measured using both creatinine and cystatin C measures, which is more accurate than measuring using either creatinine or cystatin C alone [53]. Unlike many studies that have assessed kidney function solely based on eGFR or creatinine levels [6, 32], our study also employed albuminuria criteria, allowing us to identify kidney dysfunction even in participants with a normal eGFR but abnormal uACR levels. The use of uACR, an independent, urine-based measure, may highlight a potential shift in the field away from the reliance on eGFR, a commonly used blood-based indicator of kidney function. While reduced eGFR may affect kidney secretion of ATN biomarkers, additional research is needed to understand how albuminuria affects these markers. By also focusing on uACR, our study offers an alternative to the eGFR-only approach, potentially paving the way for new insights into the importance of other CKD markers and their role in dementia risk among diverse populations. In this study, we examined eGFR and uACR independently; however, future studies comparing the two methods (i.e., eGFR or uACR alone versus eGFR + uACR) are warranted. Determining the concordance of CKD with continuous eGFR and/or uACR measures would also be beneficial.

### Limitations

There are limitations to this study. This study was cross-sectional; additional studies are needed to examine the longitudinal impact of kidney function on ATN biomarkers and the clinical implications. It is important to note that most individuals with CKD in this study had moderate disease (see Supplemental Table 5). Severe CKD can exhibit more pronounced effects on ATN biomarkers, as shown in a previous study [54]. The current study population included relatively few individuals undergoing dialysis (*n* = 14), and as such, a subgroup analysis was not performed. Liu et al. reported that individuals with CKD undergoing dialysis had lower serum Aβ42 and Aβ40 levels compared to those not receiving dialysis, with levels similar to those observed in healthy controls [55]. That study did not calculate the Aβ42/40 ratio, nor did they measure other ATN markers; thus, future studies are needed to determine the potential effects of dialysis on these biomarkers. The current study did not stratify participants by cognitive status (i.e., cognitively unimpaired, mild cognitive impairment, or dementia). At present, only MCI status is available; adjudication of cognitive impairment and dementia is ongoing as part of the third study visit. Future analyses will consider stratification by cognitive status to better contextualize blood-based biomarker levels. Gold-standard AD biomarkers such as cerebrospinal fluid (CSF) and positron emission tomography (PET) imaging were not available in this study, limiting pathological validation.

## Conclusion

Overall, this study found that CKD was associated with higher plasma levels of p-Tau181, NfL, and GFAP, and lower Aβ42/Aβ40 ratio in a diverse study of middle-aged and older Hispanic/Latino individuals. Lower eGFR levels (i.e., higher kidney dysfunction) and higher uACR (i.e., albuminuria) were associated with higher levels of p-Tau181, NfL, and GFAP, and lower Aβ42/Aβ40 ratio. Additionally, the associations between kidney function measures and plasma ATN biomarkers were independent of diabetes and hypertension, which are the primary cardiometabolic risk factors for kidney dysfunction. These findings suggest that the presence of CKD, defined by eGFR < 60 ml/min/1.73m^2^ or albuminuria, can confound plasma ATN biomarker concentrations, which may have implications for interpretation in dementia research and clinical practice.

## Electronic supplementary material

Below is the link to the electronic supplementary material.

Supplementary Material 1

## Data Availability

The datasets supporting the conclusions of this article are not readily available. However, requests to access the data from the Hispanic Community Health Study/Study of Latinos (HCHS/SOL) and SOL-Investigation of Neurocognitive Aging (SOL-INCA) can be directed to the Biologic Specimen and Data Repository Information Coordinating Center (BioLINCC) at https://biolincc.nhlbi.nih.gov/studies/hchssol/.
