# Supplementary material for: Kidney function is associated with plasma ATN biomarkers among Hispanics/Latinos: SOL-INCA and HCHS/SOL results
Source: Alzheimers Res Ther. 2025 Jun 19;17:137. doi: 10.1186/s13195-025-01786-8 (PMC12180232; doi:10.1186/s13195-025-01786-8)
Supplement: Supplementary file 1 — Supplementary Material 1 [file 13195_2025_1786_MOESM1_ESM.docx]

**Online-Only Supplementary Materials**

This document includes the following supplementary elements:

- **Supplemental Table 1.** Associations of eGFR and uACR with plasma biomarkers in the Study of Latinos – Investigation of Neurocognitive Aging (SOL-INCA, N= 5,968)
- **Supplemental Table 2.** Associations of low eGFR (eGFR<60 ml/min per 1.73 m2) and albuminuria (uACR>=30 mg/g) with plasma biomarkers in the Study of Latinos – Investigation of Neurocognitive Aging (SOL-INCA, N= 5,968)
- **Supplemental Table 3.** Associations of chronic kidney disease with NfL in the multiplex subsample in the Study of Latinos – Investigation of Neurocognitive Aging (SOL-INCA, N= 5,791)
- **Supplemental Table 4.** Associations of chronic kidney disease with plasma biomarkers excluding outliers at 3 standard deviations above and below the mean in the Study of Latinos – Investigation of Neurocognitive Aging (SOL-INCA, N= 5,968)
- **Supplemental Figure 1.** Associations of eGFR and uACR with plasma biomarkers in the Study of Latinos – Investigation of Neurocognitive Aging (SOL-INCA, N= 5,968)
- **Supplemental Table 5.** Number of participants based on CKD risk category, eGFR category, and uACR category in the Study of Latinos – Investigation of Neurocognitive Aging (SOL-INCA, N= 5,968).

**Supplemental Table 1.** Associations of eGFR and uACR with plasma biomarkers in the *Study of Latinos – Investigation of Neurocognitive Aging* (SOL-INCA, N= 5,968)

|  | **eGFR** | | **(Ln) uACR** | |
| --- | --- | --- | --- | --- |
|  | **M1** | **M2** | **M1** | **M2** |
| **Outcomes** | **b [CI 95%]** | **b [CI 95%]** | **b [CI 95%]** | **b [CI 95%]** |
| Aβ42/40 | 0.000*** [0.000;0.000] | 0.000*** [0.000;0.000] | -0.001** [-0.001;-0.000] | -0.001*** [-0.001;-0.000] |
| pTau-181 | -0.026*** [-0.031;-0.022] | -0.026*** [-0.031;-0.022] | 0.235*** [0.173;0.297] | 0.236*** [0.175;0.298] |
| NfL | -0.335*** [-0.383;-0.287] | -0.328*** [-0.378;-0.279] | 3.038*** [2.402;3.673] | 3.340*** [2.712;3.968] |
| GFAP | -0.840*** [-0.988;-0.692] | -0.872*** [-1.015;-0.730] | 4.736*** [2.248;7.224] | 5.492*** [3.055;7.930] |

*Note*. All reported values account for the complex survey designs of the SOL-INCA. eGFR=estimated glomerular filtration rate, uACR= urine albumin creatinine ratio, Aβ =beta-amyloid, pTau=phosphorylated tau, NfL=neurofilament light, GFAP=glial fibrillary acidic protein, b=beta, CI=confidence intervals. ^*^*p* <0.05, ^**^*p* <0.01, ^***^*p* <0.001. uACR was natural log-transformed prior to modeling. Each of the plasma biomarker outcomes was independently modeled as a function of each exposure (eGFR, uACR). M1 adjusted for age, sex, Hispanic/Latino background, apolipoprotein E genotype (APOE), body mass index, glycosylated hemoglobin, systolic blood pressure, and diastolic blood pressure. M2 adjusted for age, sex, Hispanic/Latino background, APOE, body mass index, diabetes, and hypertension.

**Supplemental Table 2.** Associations of low eGFR (eGFR<60 ml/min per 1.73 m2) and albuminuria (uACR>=30 mg/g) with plasma biomarkers in the Study of Latinos – Investigation of Neurocognitive Aging (SOL-INCA, N= 5,968)

|  | **Low eGFR (eGFR<60 ml/min per 1.73 m2)** | | **Albuminuria (uACR>=30 mg/g)** | |
| --- | --- | --- | --- | --- |
|  | **M1** | **M2** | **M1** | **M2** |
| **Outcomes** | **b [CI 95%]** | **b [CI 95%]** | **b [CI 95%]** | **b [CI 95%]** |
| Aβ42/40 | -0.004** [-0.006;-0.001] | -0.004** [-0.007;-0.001] | -0.004*** [-0.005;-0.002] | -0.004*** [-0.006;-0.002] |
| pTau-181 | 1.728*** [1.346;2.109] | 1.798*** [1.402;2.194] | 0.678*** [0.475;0.881] | 0.723*** [0.520;0.927] |
| NfL | 22.598*** [18.791;26.405] | 23.239*** [19.291;27.187] | 9.586*** [7.337;11.834] | 11.135*** [8.799;13.471] |
| GFAP | 54.709*** [41.394;68.024] | 58.347*** [45.139;71.555] | 21.394*** [12.022;30.767] | 23.797*** [14.418;33.175] |

*Note*. All reported values account for the complex survey designs of the SOL-INCA. eGFR=estimated glomerular filtration rate, uACR= urine albumin creatinine ratio, Aβ =beta-amyloid, pTau=phosphorylated tau, NfL=neurofilament light, GFAP=glial fibrillary acidic protein, b=beta, CI=confidence intervals. ^*^*p* <0.05, ^**^*p* <0.01, ^***^*p* <0.001. Each of the plasma biomarker outcomes was independently modeled as a function of each exposure. M1 adjusted for age, sex, Hispanic/Latino background, apolipoprotein E genotype (APOE), body mass index, glycosylated hemoglobin, systolic blood pressure, and diastolic blood pressure. M2 adjusted for age, sex, Hispanic/Latino background, APOE, body mass index, diabetes, and hypertension.

**Supplemental Table 3.** Associations of chronic kidney disease with NfL in the multiplex subsample in the Study of Latinos – Investigation of Neurocognitive Aging (SOL-INCA, N= 5,791)

|  | **M1** | **M2** |
| --- | --- | --- |
| **Outcomes** | **b [CI 95%]** | **b [CI 95%]** |
| NfL | 10.391*** [8.485;12.298] | 11.727*** [9.717;13.736] |

Note. All reported values account for the complex survey designs of the SOL-INCA. NfL=neurofilament light, b=beta, CI=confidence intervals. *p <0.05, **p <0.01, ***p <0.001. M1 adjusted for age, sex, Hispanic/Latino background, apolipoprotein E genotype (APOE), body mass index, glycosylated hemoglobin, systolic blood pressure, and diastolic blood pressure. M2 adjusted for age, sex, Hispanic/Latino background, APOE, body mass index, diabetes, and hypertension.

**Supplemental Table 4.** Associations of chronic kidney disease with plasma biomarkers excluding outliers at 3 standard deviations above and below the mean in the Study of Latinos – Investigation of Neurocognitive Aging (SOL-INCA, N= 5,968)

|  | **M1** | **M2** |
| --- | --- | --- |
| **Outcomes** | **b [CI 95%]** | **b [CI 95%]** |
| Aβ42/40 | -0.003*** [-0.005;-0.002] | -0.004*** [-0.006;-0.002] |
| pTau-181 | 0.574*** [0.441;0.706] | 0.587*** [0.456;0.718] |
| NfL | 7.522*** [6.300;8.744] | 8.558*** [7.313;9.803] |
| GFAP | 27.297*** [19.072;35.523] | 29.552*** [21.350;37.755] |

*Note*. All reported values account for the complex survey designs of the SOL-INCA. Aβ =beta-amyloid, pTau=phosphorylated tau, NfL=neurofilament light, GFAP=glial fibrillary acidic protein, b=beta, CI=confidence intervals. ^*^*p* <0.05, ^**^*p* <0.01, ^***^*p* <0.001. Each of the plasma biomarker outcomes was independently modeled as a function of chronic kidney disease. Extreme values at 10 standard deviations above and below the mean of the plasma biomarkers were excluded prior to the original analyses. M1 adjusted for age, sex, Hispanic/Latino background, apolipoprotein E genotype (APOE), body mass index, glycosylated hemoglobin, systolic blood pressure, and diastolic blood pressure. M2 adjusted for age, sex, Hispanic/Latino background, APOE, body mass index, diabetes, and hypertension.

**Supplemental Figure 1.** Associations of eGFR and uACR with plasma biomarkers in the *Study of Latinos – Investigation of Neurocognitive Aging* (SOL-INCA, N= 5,968)


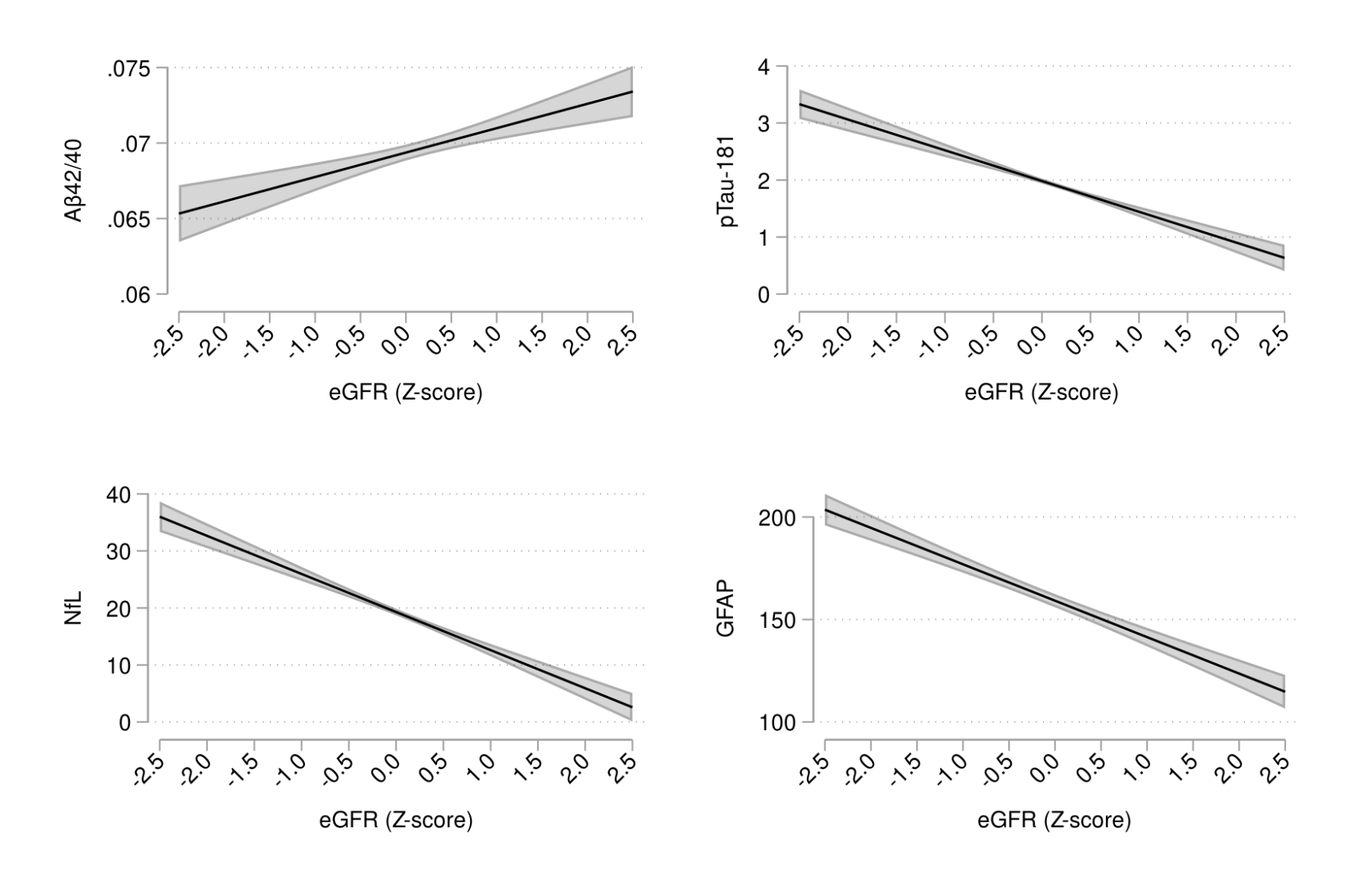
(i) eGFR

(ii) uACR


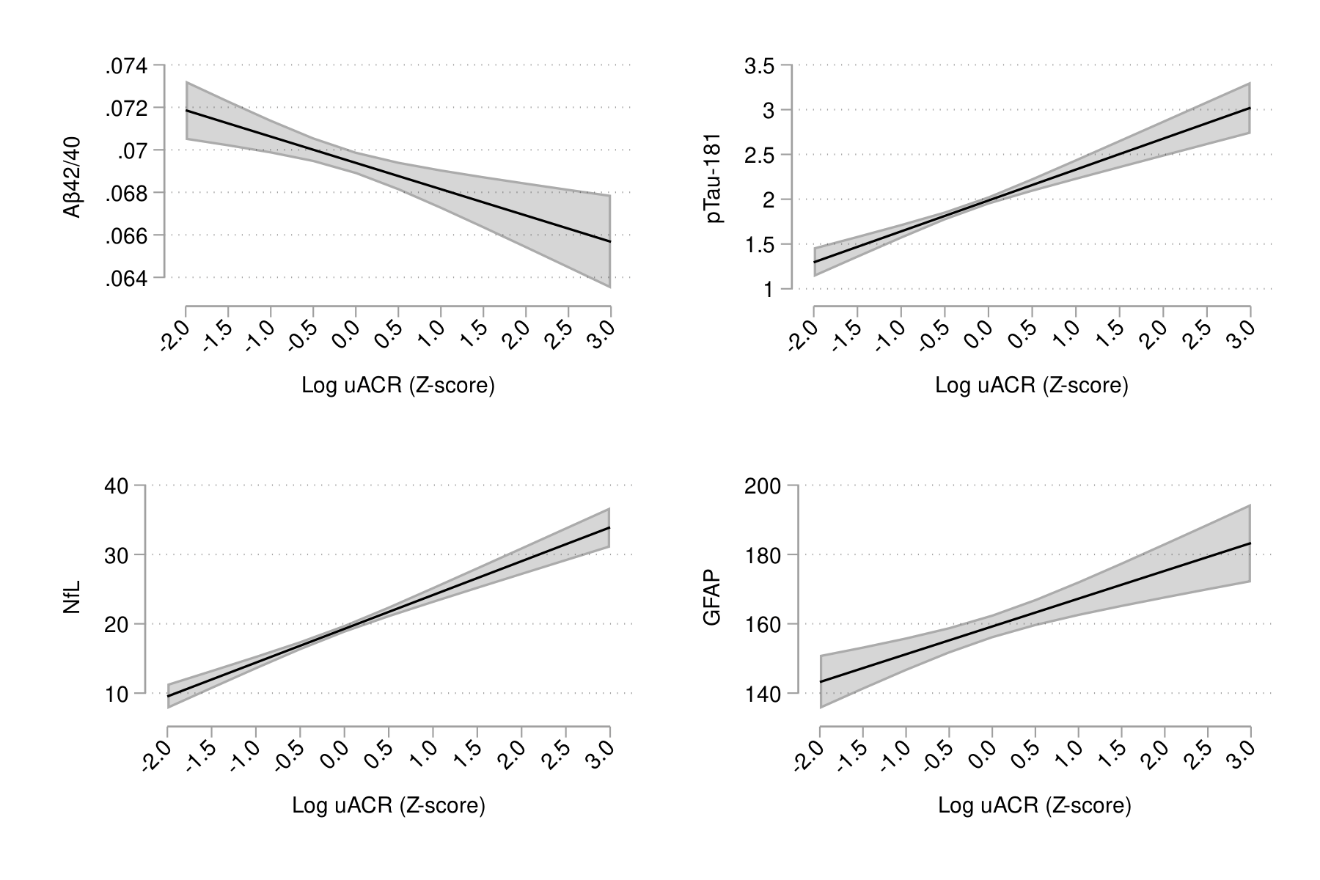


*Note*. eGFR=estimated glomerular filtration rate, uACR= urine albumin creatinine ratio, Aβ =beta-amyloid, pTau=phosphorylated tau, NfL=neurofilament light, GFAP=glial fibrillary acidic protein. uACR was natural log-transformed prior to modeling (range from -0.97 to 10.2). The model adjusted for age, sex, Hispanic/Latino background, apolipoprotein E (APOE) genotype, body mass index, diabetes, and hypertension.

**Supplemental Table 5.** Number of participants based on CKD risk category, eGFR category, and uACR category in the *Study of Latinos – Investigation of Neurocognitive Aging* (SOL-INCA, N= 5,968).

| **CKD risk category** | unweighted N | weighted % |
| --- | --- | --- |
| Low | 5041 | 82.8 |
| Moderate | 634 | 11.3 |
| High | 174 | 3.5 |
| Very high | 119 | 2.4 |
| **Total** | **5968** | **100.0** |
| (corresponds to Green, Yellow, Orange, Red, Prognosis of CKD by GFR and Albuminuria Categories KDIGO 2012) | | |
|  |  |  |
| **eGFR category** | unweighted N | weighted % |
| Normal (egfr>=90) | 3848 | 60.9 |
| Mild (egfr>=60, egfr<90) | 1779 | 32.4 |
| Mild to Moderate (egfr>=45, egfr<60) | 207 | 4.0 |
| Moderate to Severe (egfr>=30, egfr<45) | 83 | 1.7 |
| Severe (egfr>=15, egfr<30) | 33 | 0.7 |
| Kidney failure (egfr<15) | 18 | 0.3 |
| **Total** | **5968** | **100.0** |
| (corresponds to G1, G2, G3a, G3b, G4, G5) | | |
|  |  |  |
| **uACR category** | unweighted N | weighted % |
| Normal (uacr<30) | 5216 | 86.2 |
| Moderate (uacr>=30, uacr<=300) | 593 | 10.7 |
| Severe (uacr>300) | 159 | 3.1 |
| **Total** | **5968** | **100.0** |
| (corresponds to A1, A2, A3) | | |

Note: Prevalence estimates (%) were weighted to allow generalizations to the target population of the SOL-INCA study.
